# Intracellular Trafficking Mechanisms of Synaptic Dysfunction in Alzheimer’s Disease

**DOI:** 10.3389/fncel.2020.00072

**Published:** 2020-04-17

**Authors:** Catarina Perdigão, Mariana A. Barata, Margarida N. Araújo, Farzaneh S. Mirfakhar, Jorge Castanheira, Cláudia Guimas Almeida

**Affiliations:** Laboratory Neuronal Trafficking in Aging, CEDOC Chronic Diseases Research Center, NOVA Medical School, Universidade NOVA de Lisboa, Lisbon, Portugal

**Keywords:** late-onset Alzheimer’s disease, synapses, endocytosis, β-amyloid, APOE4, PICALM, BIN1, CD2AP

## Abstract

Alzheimer’s disease (AD) is the most common neurodegenerative disease characterized by progressive memory loss. Although AD neuropathological hallmarks are extracellular amyloid plaques and intracellular tau tangles, the best correlate of disease progression is synapse loss. What causes synapse loss has been the focus of several researchers in the AD field. Synapses become dysfunctional before plaques and tangles form. Studies based on early-onset familial AD (eFAD) models have supported that synaptic transmission is depressed by β-amyloid (Aβ) triggered mechanisms. Since eFAD is rare, affecting only 1% of patients, research has shifted to the study of the most common late-onset AD (LOAD). Intracellular trafficking has emerged as one of the pathways of LOAD genes. Few studies have assessed the impact of trafficking LOAD genes on synapse dysfunction. Since endocytic traffic is essential for synaptic function, we reviewed Aβ-dependent and independent mechanisms of the earliest synaptic dysfunction in AD. We have focused on the role of intraneuronal and secreted Aβ oligomers, highlighting the dysfunction of endocytic trafficking as an Aβ-dependent mechanism of synapse dysfunction in AD. Here, we reviewed the LOAD trafficking genes APOE4, ABCA7, BIN1, CD2AP, PICALM, EPH1A, and SORL1, for which there is a synaptic link. We conclude that in eFAD and LOAD, the earliest synaptic dysfunctions are characterized by disruptions of the presynaptic vesicle exo- and endocytosis and of postsynaptic glutamate receptor endocytosis. While in eFAD synapse dysfunction seems to be triggered by Aβ, in LOAD, there might be a direct synaptic disruption by LOAD trafficking genes. To identify promising therapeutic targets and biomarkers of the earliest synaptic dysfunction in AD, it will be necessary to join efforts in further dissecting the mechanisms used by Aβ and by LOAD genes to disrupt synapses.

## Introduction

Alzheimer’s disease (AD), the most common neurodegenerative disease, affecting 1 in 10 people over 65 years old, remains without adequate treatment. By the age of 85, one in three people develops the disease (Alzheimer’s Association, 2019). Functionally, a progressive loss of memory and cognitive impairment leads to a loss of autonomy and independance. Neuropathologically, AD is characterized by two hallmark proteinaceous aggregates, extracellular amyloid plaques and intracellular neurofibrillary tangles. The AD brain volume is dramatically reduced, mainly due to extensive cortical neuronal death. These are hallmark phenotypes of a disease in which pathological mechanisms can start 30 years before diagnosis. Importantly, the synapse loss is the best pathological correlate of cognitive impairment in AD, which can exceed and precede the existing neuronal cell death (Tampellini and Gouras, 2010). Among the earliest disease phenotypes are intracellular β-amyloid (Aβ) accumulation and synapse dysfunction. Synapse dysfunction can remain silent, at least initially, due to the reserve brain capacity to compensate for the affected neurons. Cognitive impairment likely manifests when the pathology spreads throughout the brain. Thus, it has become apparent that the next step for AD research is to focus on identifying novel therapeutic strategies targeted at the earliest mechanisms of synaptic dysfunction.

AD has two forms (Katzman, 1976), the familial early-onset (eFAD) and sporadic late-onset (LOAD), depending on if it occurs before or after the age of 65. eFAD is the result of dominantly inherited mutations in the amyloid precursor protein (APP) and presenilin (PSEN1 and PSEN2; Selkoe and Hardy, 2016). These mutations lead to increased production of Aβ, the main component of amyloid plaques, or a higher ratio of longer Aβ peptides (Aβ42/43) to Aβ40. Aβ42, while accounting for only 10% of the total Aβ produced, is more hydrophobic and has a higher capacity of aggregating into oligomers. Currently, evidence strongly supports that soluble Aβ oligomers are more toxic than insoluble fibrils (plaques). The production of Aβ42 occurs intracellularly, where it accumulates before being secreted and deposited into amyloid plaques extracellularly (Gouras et al., 2005). Aβ oligomerization, particularly of Aβ42 peptides, begins intracellularly (Tampellini and Gouras, 2010). Intraneuronal Aβ correlates with cognitive dysfunction better than amyloid plaques (Billings et al., 2005; Takahashi et al., 2017). Aβ42 oligomerization precedes tau aggregation (Billings et al., 2005; Bilousova et al., 2016). Thus, we did not include tau-dependent synaptic dysfunction in this review; nevertheless, since tau aggregation is a determinant for the propagation and progression of the pathology, we recommend a recent review on the topic (Tracy and Gan, 2018).

**Figure 1:**
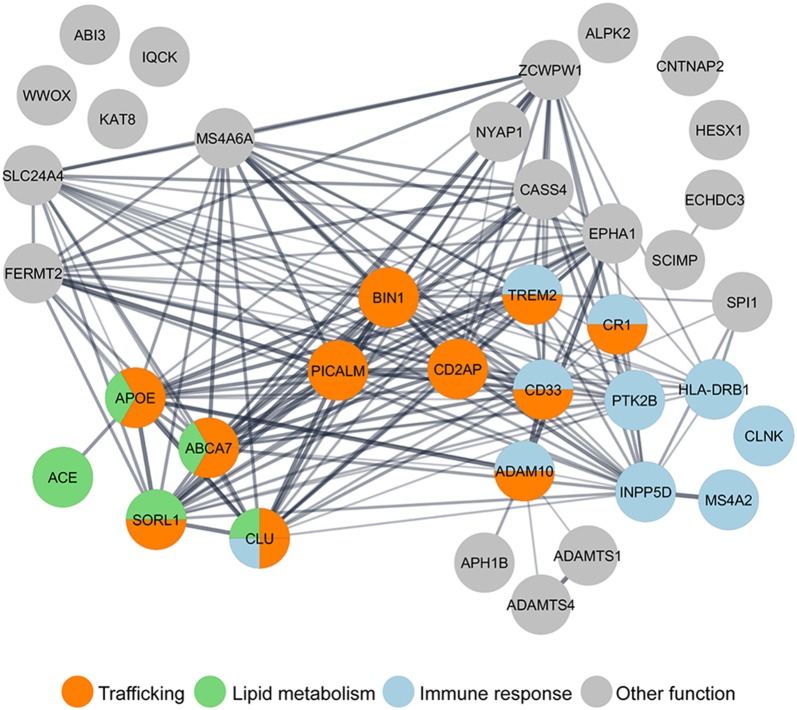
Gene ontology (GO) analysis of LOAD risk factors reveals the enrichment of genes in the trafficking pathway. Nearly half of the 37 LOAD putative risk factors identified by genome-wide association studies (GWAS) meta-analysis (Lambert et al., 2013; Jansen et al., 2019; Kunkle et al., 2019), have been functionally grouped by GO analysis in three main pathways: trafficking (orange), immune response (blue), and lipid metabolism (green). GO analysis was performed using the Cytoscape StringApp (Doncheva et al., 2019).

In contrast with eFAD, LOAD is likely multifactorial, caused by a combination of aging, lifestyle, and genetic factors. Given the prediction for heritability of AD to be between 58 and 79%, geneticists have been looking for gene variants in LOAD patients (Gatz et al., 2006). Apolipoprotein E-ε4 was the first genetic risk factor identified and is the most significant one (Strittmatter et al., 1993). However, it does not account for all genetic susceptibility (Stocker et al., 2018). Thus, genome-wide association studies (GWAS) conducted since 2007 have identified several additional genetic risk factors associated with LOAD (Grupe et al., 2007; Harold et al., 2009; Hollingworth et al., 2011; Naj et al., 2014). A recent meta-analysis of GWAS expanded the initial “Top 10” genes (Lambert et al., 2013,^1^) to 37 LOAD putative risk factors (Jansen et al., 2019; Kunkle et al., 2019). Now it is crucial to determine how these genes contribute to AD functionally. We, along with other resesarchers, have performed gene ontology (GO) analysis and LOAD risk factors group in three main pathways: endocytic trafficking, immune response, and lipid metabolism (Figure 1). Interestingly, some genes, such as ABCA7 and TREM2, are grouped in more than one pathway. Indeed, it is essential to keep in mind that these biological pathways intersect; for example, lipid metabolism influences trafficking at many levels (Huijbregts et al., 2000) and trafficking in microglia influences the immune response (McQuade and Blurton-Jones, 2019). Most attention has been given to how the loss of function of these genes impacts Aβ, generation (reviewed in Guimas Almeida et al., 2018), aggregation (Zhang et al., 2018), and clearance (Van Acker et al., 2019). Recently reviewed data indicates that LOAD microglia risk genes may impact synapses (Rajendran and Paolicelli, 2018).

Here, we will review the importance of neuronal endocytic trafficking for synapses (Barry and Ziff, 2002), how Aβ can have a physiological synaptic role, and how intraneuronal and secreted Aβ oligomers affect synapses, highlighting Aβ-dependent consequences to synaptic endocytic trafficking. Importantly, we will highlight recent data on how LOAD risk genes related to trafficking may impact synapse dysfunction, in particular, the ones related to endocytic protein trafficking (BIN1, SORL1, PICALM, CD2AP), to lipid trafficking (APOE and ABCA7), and synapses (EPH1A).

## Synaptic Endocytic Trafficking

### Presynaptic Trafficking

Cycles of synaptic vesicles (SV) exocytosis and endocytosis at the presynaptic active zone membrane mediate neurotransmitter release. This presynaptic trafficking begins with the arrival of an action potential that opens Ca^2+^ channels, which triggers exocytosis of readily releasable SV pool (RRP), which comprises the SVs that are docked and primed for exocytosis. There is also the SV reserve pool, which is mobilized only during periods of intense neuronal activity. After exocytosis, there are different routes of endocytosis to recycle SVs. SV components and newly added membrane need to be recycled to ensure repeated rounds of release.

The SV fusion machinery is composed of the SNARE (soluble N-ethylmaleimide-sensitive factor attachment protein receptor) complex proteins synaptobrevin/VAMP2, syntaxin-1, and SNAP-25 and SM proteins (“Sec1/Munc-18-like proteins”), which promote the assembly of the fusogenic SNARE complex. To trigger exocytosis, Ca^2+^ binds to synaptotagmin-1, the SV calcium sensor protein that, by interacting with the SNARE complex, modulates its assembly or its coupling to the plasma membrane, enabling fusion (Tucker et al., 2004; Wang et al., 2011; Dittman and Ryan, 2019). Upon fusion, NSF promotes the disassembly and recycling of the SNARE complex (Südhof, 2013). SV recycling follows exocytosis to regulate the presynaptic plasma membrane area and locally regenerate SVs (Kononenko and Haucke, 2015; Azarnia Tehran et al., 2018; Chanaday et al., 2019). There are probably several routes of SV recycling. Here, we focus on endocytic recycling that starts with endocytosis of SV proteins. When SVs fuse, they intermix with the plasma membrane (Fernández-Alfonso et al., 2006); therefore, SV recycling requires sorting and reformation. Considering the high number of SVs, their identity and uniformity, protein sorting, and SV reformation are significant tasks for the presynaptic endocytic machinery. Presynaptic endocytosis can be mediated by clathrin, by bulk endocytosis, or by ultrafast endocytosis. Upon the formation of the presynaptic early endosome, SVs need to be accurately reformed, which requires endocytic adaptors such as the assembly protein complex 2 (AP-2) to sort cargo into clathrin-coated pits. AP-3 is an alternative adaptor that can sort SV cargo at endosomes into reforming SVs but independently of clathrin (Milosevic, 2018). Other clathrin adaptors are neuronal-specific such as AP180 and LOAD risk factor CALM (for LOAD risk factors, see below). These proteins are essential for VAMP2 sorting by still unclear mechanisms. Other endocytic adaptors are required to ensure sorting fidelity, such as stonin 2 and synaptic vesicle 2 (SV2A; Kononenko and Haucke, 2015; Gordon and Cousin, 2016). After the assembly of endocytic vesicles, the BAR domain proteins, endophilin, amphiphysin, and SNX9/18 form the vesicle neck and recruit dynamin, which is responsible for membrane scission. Then, endophilin recruits synaptojanin-1 to shed the coat off of the released endocytic vesicles (Kononenko and Haucke, 2015).

### Postsynaptic Trafficking

In the postsynaptic site, activation of neurotransmitter-gated ion channels occurs by the presynaptic release of neurotransmitters. In an excitatory synapse, the principal neurotransmitter released is glutamate, which binds NMDA (N-methyl-D-aspartic acid) receptors, kainate receptors, and AMPA (α-amino-3-hydroxy-5-methyl-4-isoxazole propionic acid) receptors (Waites et al., 2005). Brief periods of high neuronal excitability activate NMDARs and induce Ca^2+^ influx, leading to a long-lasting increase in synaptic efficacy. Repetitive low-frequency stimulation produces long-term depression (LTD), with a decrease in synaptic strength (Kessels and Malinow, 2009). Plasticity at synapses, i.e., changes in the onset or magnitude of long-term potentiation (LTP) and LTD, can be regulated postsynaptically by changing the number, types, or properties of neurotransmitter receptors (reviewed in Henley and Wilkinson, 2016). Regulated trafficking of AMPARs underlies activity-induced changes in synaptic transmission, and therefore their abundance at synapses can significantly strengthen or weaken synaptic transmission (Malinow and Malenka, 2002; Bredt and Nicoll, 2003; Newpher and Ehlers, 2008). AMPAR subunits can have long- (GluA1, GluA2L, and GluA4) or short-tailed carboxyl-terminals (GluA2, GluA3, and GluA4S), which impact their trafficking (Shepherd and Huganir, 2007; Hanley, 2008). The recycling or functional insertion of AMPARs into the postsynaptic membrane is dependent on AMPAR-binding proteins, phosphorylation, and ubiquitination events (Derkach et al., 1999, 2007; Malinow and Malenka, 2002; Schwarz et al., 2010). The addition of long-tailed AMPARs to the postsynaptic membrane is correlated with synaptic strengthening and, therefore, with LTP (Hayashi et al., 2000; Kakegawa et al., 2004), while LTD is associated with the removal of synaptic long- or short-tailed AMPARs (Malinow and Malenka, 2002; Sheng and Hyoung Lee, 2003).

### Aβ Physiological Role at Synapses

Although in AD, high Aβ concentration has a toxic effect, endogenous Aβ concentration has a physiological synaptic role. Aβ physiological function was first proposed in 2003 by the Malinow lab and described that blocking Aβ endogenous production by gamma-secretase inhibition potentiated synaptic transmission (Kamenetz et al., 2003). In contrast, in 2008, the Arancio lab showed that exogenous picomolar concentrations of Aβ, monomers and oligomers, increase LTP, while high nanomolar amounts of Aβ reduce LTP (Puzzo et al., 2008). In agreement, the Slutsky lab showed that increasing endogenous Aβ, by inhibition of neprilysin-mediated degradation, potentiates synaptic transmission (Abramov et al., 2009). Importantly, SV recycling was first identified to be endogenously regulated by Aβ. The Slutsky lab elegantly demonstrated that increasing Aβ by neprilysin inhibition increases SV recycling, while decreasing physiological Aβ levels by anti-Aβ antibody-promoted degradation, in contrast, decreases SV recycling (Abramov et al., 2009). More recent data indicate that exogenous picomolar preparations of oligomeric Aβ42 can augment neurotransmitter release and the length of the postsynaptic density, resulting in a late-LTP (Gulisano et al., 2019). The mechanisms of how endogenous Aβ regulates synaptic vesicle recycling and PSD recruitment for modulating synaptic transmission and plasticity are unclear. Some evidence indicates that Aβ can bind to alpha7-nicotinic acetylcholine receptors (Puzzo et al., 2008; Gulisano et al., 2019) to induce presynaptic calcium entry. Overall, the evidence points to an Aβ physiological role, but it is still not clear which form of Aβ is relevant. Does Aβ40 being more abundant play a different physiological role from Aβ42? Other products of APP processing, as well as APP full length, also have synaptic physiological functions that have been recently reviewed (Ludewig and Korte, 2016).

### Aβ-Direct Impact on Synaptic Trafficking

There are several lines of evidence demonstrating that Aβ is synaptotoxic. In most experimental conditions, exogenous preparations of synthetic or brain-derived oligomeric Aβ were used (reviewed by Mucke and Selkoe, 2012), while other experimental conditions mimicked chronic and time-dependent Aβ accumulation with overexpression of mutant APP, with only a few assessing the contribution of intracellular Aβ. Here we will cover intracellular Aβ-dependent mechanisms focusing on trafficking dysfunction.

Aβ progressively accumulates within neurons (Gouras et al., 2000; Mochizuki et al., 2000) in the limiting membrane of late endosomes or MVBs pre- and especially postsynaptically (Takahashi et al., 2002; Willén et al., 2017; Yu et al., 2018). This intracellular Aβ can oligomerize and disrupt synapses (Takahashi et al., 2004; Pickett et al., 2016). Increased Aβ production, as a result of overexpression of exogenous APP or β-CTF in hippocampal slices, is sufficient to depress synaptic transmission, since inhibiting beta- or gamma-secretase prevented synaptic depression (Kamenetz et al., 2003).

More mechanistically, increased Aβ42 production, as a result of overexpression of APP with eFAD Swedish mutation (APPsw) in primary neurons, is enough to trigger postsynaptic dysfunction with loss of PSD-95, AMPA and NMDA glutamate receptors (Almeida et al., 2005; Snyder et al., 2005). Loss of PSD-95 and spines also occurs *in vivo* in AD mice and human AD brain (Gylys et al., 2004; Pham et al., 2010; Proctor et al., 2010; Sultana et al., 2010; Baglietto-Vargas et al., 2018). Since PSD-95 drives AMPA receptors’ incorporation in the postsynaptic density, its loss may underlie the synaptic removal of these receptors (Ehrlich and Malinow, 2004). The loss of AMPA receptors likely causes the reduced AMPA receptor-mediated currents observed even when APP is overexpressed (Ting et al., 2007). Aβ42 requirement for loss of AMPA transmission was confirmed when a mutation that inhibits BACE1 cleavage (APPM596V) blocked synaptic depression (Ting et al., 2007). Indeed, Aβ-dependent synaptic endocytosis of AMPA receptors is sufficient to account for spine loss and reduced NMDA synaptic response (Hsieh et al., 2006). One interesting study found that intracellular Aβ oligomerization, induced by overexpression of APP with the Osaka mutation, reduced spines *via* dysfunction of BDNF, mitochondria, and endosomes transport (Umeda et al., 2015). Intracellular Aβ also interferes with the BDNF TrkB receptors’ endosomal sorting for lysosomal degradation, which could disturb synapses (Almeida et al., 2006).

The presynaptic compartment may get affected after the postsynaptic compartment since the loss of synaptophysin, a major component of SVs, only occurred after AMPA receptor synaptic loss (Almeida et al., 2005). Indeed, the presynaptic decrease of synaptophysin and synapsin mark AD synaptic loss. Interestingly, the presynaptic compartments of APPsw neurons are enlarged but undergo SV recycling (Almeida et al., 2005). Also, upon sustained neuronal activation of APPwt neurons, SV recycling is reduced (Ting et al., 2007). The defects in SV endocytosis could be partially due to dynamin-1 depletion induced by APPsw overexpression in eFAD mice (Kelly et al., 2005; Parodi et al., 2010). The contribution of intracellular Aβ to SV cycle dysfunction remains mostly unstudied.

Secreted Aβ can affect synapses extracellularly and by contributing to intracellular Aβ *via* endocytosis of extracellular Aβ (Lai and McLaurin, 2010). Endocytosed Aβ oligomers could translocate to synapses where interaction with the SV marker synaptophysin can be detected (Russell et al., 2012). Extracellular Aβ can also form a complex with secreted ApoE. This complex can bind to low-density lipoprotein receptor (LDLR) and LRP1, internalize, and accumulate into endosomes within synapses (Bilousova et al., 2019). It is not clear how endosomal Aβ can interact with cytosolic proteins. An answer may be provided by a study that showed that endocytosed Aβ42 could accumulate in endosomes, increasing their membrane permeability and facilitating Aβ cytosolic accumulation and neuronal toxicity (Yang et al., 1998). Moreover, a recent study demonstrated that Aβ oligomers *in vitro* could inhibit the SNARE fusion complex assembly by direct binding to syntaxin-1a (Yang et al., 2015). Besides, *in vitro*, Aβ seems to be able to block synaptophysin complex formation with VAMP2 with relevance for SV exocytosis (Russell et al., 2012). Thus, when Aβ oligomers rupture endosomes and reach the cytosol, Aβ could directly inhibit the SV cycle. This mechanism is similar to the one used by alpha-synuclein in Parkinson’s disease, where cytosolic alpha-synuclein oligomerization inhibits SNARE-mediated vesicle fusion in dopaminergic neurons (Garcia-Reitböck et al., 2010; DeWitt and Rhoades, 2013).

Secreted Aβ, or exogenous synthetic Aβ, can also bind extracellularly to excitatory synapses, namely postsynaptically (Wang et al., 2017; Willén et al., 2017), increasing calcium influx, which triggers AMPA and NMDA receptor phosphorylation leading to their removal from synapses by endocytosis (Snyder et al., 2005; Liu et al., 2010; Miñano-Molina et al., 2011; Sinnen et al., 2016; Baglietto-Vargas et al., 2018). The mechanisms of disruption of glutamate receptor trafficking induced by extracellular Aβ have been extensively reviewed (Guntupalli et al., 2016). Other synaptic receptors may also be affected by exposure to Aβ oligomers, such as EphB2, the degradation of which increases upon Aβ binding (Cissé et al., 2011). EphB2 regulates NMDAR expression and currents, for long-term plasticity in the dentate gyrus, consequently regulating memory (Cissé et al., 2011). Extracellular Aβ may also contribute to spine loss *via* F-actin disassembly (Kommaddi et al., 2018). The mechanisms of SV cycle disruption by extracellular Aβ that seem to involve calcium influx have been recently reviewed (Marsh and Alifragis, 2018).

Overall, extracellular Aβ has the same targets of intracellular Aβ. While the acute treatment with Aβ oligomers promotes synaptic receptor dysfunction, chronic treatment results in abnormal spine morphology, with the induction of long thin spines that, ultimately, cause a significant decrease in spine density (Lacor et al., 2007). Alternatively, evidence indicates that extracellular Aβ depends on intracellular Aβ for synaptotoxicity as follows: Aβ binds to APP with high affinity (Lorenzo et al., 2000; Lu et al., 2003; Lacor et al., 2004; Fogel et al., 2014; Wang et al., 2017); extracellular Aβ can promote its processing and intracellular Aβ accumulation (Tampellini et al., 2009); APP enriched at synapses can be the synaptic receptor for Aβ (Laßek et al., 2013; Del Prete et al., 2014; Fanutza et al., 2015; Montagna et al., 2017); APP KO neurons are resistant to exogenous Aβ toxicity (Wang et al., 2017); and, extracellular synthetic Aβ no longer reduces PSD-95 when Aβ production is inhibited (Tampellini et al., 2009).

*In vivo* in normal rodent hippocampus, acute exposure to Aβ dimers extracted from the AD brain reduced dendritic spine density and potently inhibited LTP and enhanced LTD (Shankar et al., 2008). Interestingly while LTD required mGluR5, spine loss required NMDA receptors (Shankar et al., 2008). In a 3xTg-AD mouse model, the chronic accumulation of Aβ correlated with the impaired synaptic insertion of GluA1-containing AMPARs during chemical LTP stimulation (Baglietto-Vargas et al., 2018). However, in some AD models (APP/PS1), a postsynaptic reduction of AMPA receptors or spine loss was not a significant phenotype; instead, the presynaptic SV release was affected (He et al., 2019). A reduction of the phosphoinositol PtdIns(4, 5)P2 (PIP2) triggered by endogenous or exogenous Aβ oligomers could account for the presynaptic deficits (Berman et al., 2008; He et al., 2019). PIP2 is a plasma membrane phosphoinositide that binds to synaptotagmin-1 on the SV to allow for its docking and fusion (Lee et al., 2010). Selective inhibition of Aβ-induced PIP2 hydrolysis *via* presynaptic mGluR5 could rescue the presynaptic release of glutamate and restore synaptic transmission in APP/PS1 mice (He et al., 2019; Kunkle et al., 2019).

**Figure 2:**
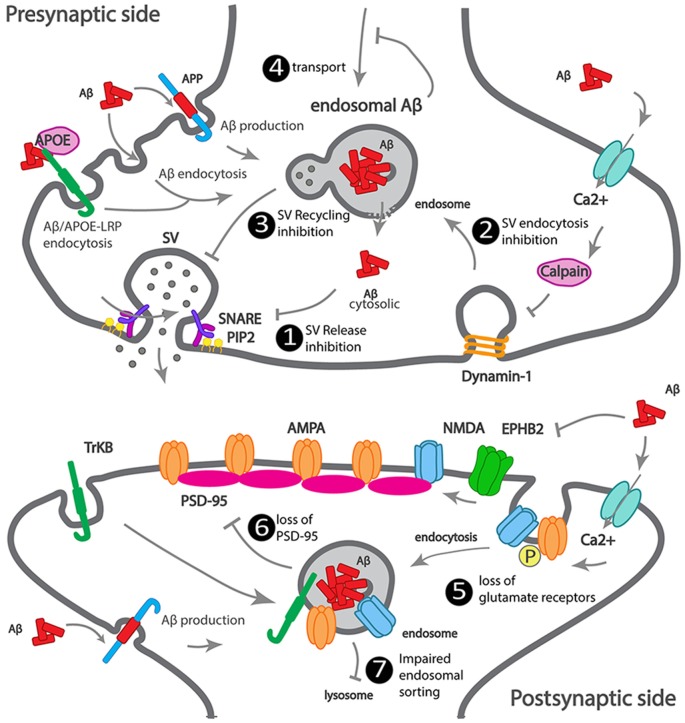
Scheme illustrating how Aβ impacts synaptic trafficking. Scheme of a dysfunctional early-onset familial AD (eFAD) synapse. At the presynaptic terminal: 1. Synaptic vesicle release inhibition, extracellular Aβ endocytosed, alone or *via* ApoE-LRP endocytosis can accumulate at endosomes. Intracellular Aβ, produced from APP endocytosis, accumulates at endosomes. Endosomal Aβ can disrupt the endosomal membrane and reach the cytosol, where it can potentially bind to SNARE subunit syntaxin-1a, or indirectly inhibit synaptophysin or PIP2, thus inhibiting synaptic vesicles (SV) fusion and neurotransmitter release. 2. SV endocytosis inhibition, extracellular Aβ increases calcium influx, triggers calcium-dependent calpain to degrade dynamin1, reducing endocytosis. 3. SV recycling inhibition by intracellular Aβ. 4. Transport to synapses inhibition by intracellular Aβ oligomerization. At the postsynaptic terminal: 5. Increased endocytosis of AMPA and NMDA receptors, by intracellular and extracellular Aβ. 6. Loss of PSD-95 by intracellular Aβ. 7. Impaired endosomal sorting of TrkB receptor, by endosomal Aβ.

We summarized how Aβ accumulates at the synapse, as well as the earliest Aβ-dependent mechanisms of synapse dysfunction (Figure 2).

### Load Genetic Risk-Driven Trafficking Defects Impact Synapses Independently of Aβ

Among the 11 LOAD risk genes involved in intracellular trafficking, we will review the evidence that supports a direct synaptic (dys)function for APOE4, ABCA7, BIN1, CD2AP, PICALM, SORL1, and EPH1A.

## APOE4

Apolipoprotein E gene (*APOE*) has a risk LOAD variant (*APOE4*). Two single nucleotide polymorphisms (SNPs) generate three allelic variants of the human APOE gene, ε2, ε3, and ε4 that affect the structure of the ApoE protein, as well as, the binding to lipids, receptors, and Aβ (Liu et al., 2013; Yamazaki et al., 2019). APOE ε3 is the most common allele, with a prevalence of 77.9%, the ε2 allele is the less prevalent (8.4%), and the ε4 allele has a frequency of 13.7% that increases in LOAD patients (Farrer et al., 1997; Michaelson, 2014).

In the brain, ApoE is the primary lipoprotein of high-density lipoprotein (HDL), mainly secreted by astrocytes and also produced by neurons (Xu et al., 1999; Hauser et al., 2011). Secreted ApoE scavenges lipids from the local environment for cellular delivery. Upon receptor-mediated endocytosis and lipid unloading, ApoE is released from its receptor at the endosomal pH and sorted for recycling or degradation (Hauser et al., 2011).

ApoE knockdown altered the cholesterol distribution within synaptic membranes (Igbavboa et al., 1997). ApoE mediates cholesterol transport into neurons, increasing synapse formation (Mauch et al., 2001; Liu et al., 2013). Cholesterol binding to synaptophysin may enable the correct sorting of SV constituents at the plasma membrane necessary for SV recycling (Thiele et al., 2000).

ApoE4 differs from ApoE3 by one aminoacid, which is sufficient to cause ApoE4 misfolding and abnormal trafficking. Reduced ApoE4 recycling and endosomal accumulation may lead to increased cholesterol intracellular accumulation (Heeren et al., 2004), as well as increased ApoE4 lysosomal degradation, accounting for the reduction of ApoE in the brain of APOE4 carriers (Riddell et al., 2008). ApoE4 is hypolipidated compared with ApoE3 and ApoE2, the most lipidated (Heinsinger et al., 2016). The APOE state of lipidation may be more critical than ApoE levels. There is some evidence that ApoE4 transports cholesterol into and out of neurons less efficiently (Michikawa et al., 2000; Rapp et al., 2006). Controversy exists since ApoE2 may delay AD development by reducing brain cholesterol (Oikawa et al., 2014).

APOE4 knock-in (KI) increased excitatory postsynaptic currents in human-induced neurons, suggesting enhanced SV release or synapse density (Lin et al., 2018). Importantly, APOE4 KI and APOE KO mice show reduced neuronal complexity and impaired synaptic plasticity (Trommer et al., 2004). Importantly, in APOE4 KI mice, LTP is diminished and LTP maintenance unimpaired (Trommer et al., 2004; Korwek et al., 2009).

Morphologically, APOE KO and APOE4 KI present less spine density and shorter spines (Wang et al., 2005; Dumanis et al., 2009; Rodriguez et al., 2013). Reduced spines could be due to both a delay in spine formation and increased spine elimination, as observed in APOE4 KI neurons *in vitro* (Nwabuisi-Heath et al., 2014). APOE4 KI show increased calcineurin activity (Neustadtl et al., 2017), a phosphatase that can dephosphorylate AMPA receptors, promoting their synaptic removal and increasing LTD (Zhou et al., 2004). Presynaptically, APOE4 KI show reduced production of the neurotransmitter glutamate (Dumanis et al., 2013) and glutamate transporter vGlut1 (Liraz et al., 2013).

ApoE receptors, members of the low-density lipoprotein (LDL) receptor family, include ApoE receptor 2 (ApoEr2), very low-density lipoprotein receptor (VLDLR), LDLR, and LDLR-related protein 1 (LRP1). ApoE receptors allow for its endocytosis and are also present at synapses, where they have specific functions (May et al., 2004; Bal et al., 2013; Bilousova et al., 2019). Presynaptically, ApoE receptors regulate SV release (Bal et al., 2013). Presynaptic activation of Apoer2 and VLDLR by Reelin, another ligand, leads to a rise in Ca^2+^ and SV fusion (Bal et al., 2013). The SNARE complex involved, containing VAMP7 and SNAP-25, was specifically up-regulated (Bal et al., 2013). Interestingly, SNAP-25 levels were higher in APOE ε4 carriers CSF compared to non-carriers with mild-cognitive impairment (MCI; Wang et al., 2018). Postsynaptically, LRP1 interaction with PSD-95 is disrupted upon NMDA activation to modulate NMDA receptors signaling (Bacskai et al., 2000; May et al., 2004). LRP1 regulates NMDA receptor function (May et al., 2004) and endocytosis, promoting, during development, the switch from NR2B to NR2A subunits at synapses (Maier et al., 2013). ApoER2 can get trapped in postsynaptic recycling endosomes upon ApoE4 binding, thus preventing reelin dependent activation of the NMDA receptor, decreasing calcium influx and LTP (Chen et al., 2010; Yong et al., 2014). Long-term spatial memory could require LDLR binding to ApoE4 (Johnson et al., 2014).

Interestingly, clusterin (or apolipoprotein J), another putative LOAD genetic risk factor, accumulates in synapses of human post-mortem brains of APOE4 AD carriers (Jackson et al., 2019).

Research is still needed to address if the detrimental synaptic effects of ApoE4 are independent of ApoE4 impact on Aβ brain accumulation. Importantly, genetically correcting APOE4 to APOE3 in LOAD IPSCs reduced synaptic release and synaptic density without however rescuing secreted Aβ (Lin et al., 2018). Complementarity may also exist since APOE KO or APOE4 KI can potentiate LTP inhibition induced by Aβ oligomers (Trommer et al., 2005). Also, APOE4 KI in an early onset model (5xTg mice) increases amyloid pathology and enhances age-dependent decline in cognitive function by down-regulating an NMDA receptor pathway (Liu et al., 2015).

## ABCA7

ATP-binding cassette transporter A7** (**ABCA7) belongs to the ABC transporter family that transports lipids, including cholesterol and lipophilic proteins, across membranes. While some ABCA7 variants increase the risk of developing AD (Carrasquillo et al., 2015; Cuyvers et al., 2015; Steinberg et al., 2015; Allen et al., 2017), others are protective (Vasquez et al., 2013). In LOAD, it is not clear whether ABCA7 expression is altered early in the disease; surprisingly, in advanced AD, ABCA7’s higher expression was associated with advanced cognitive decline (Karch et al., 2012). ABCA7 knockout mice have altered brain lipid profiles, and when aged, show impaired spatial memory (Sakae et al., 2016) with more pronounced effects in female mice (Logge et al., 2012). ABCA7 knockout may accelerate amyloid pathology in some eFAD mouse models (APP/PS1; Sakae et al., 2016), but not in others (J20; Kim et al., 2013). Whether or not ABCA7 knockout impacts cognitive function needs to be studied. Suggestive of an effect on synaptic function, a recent study found that healthy ABCA7 carriers show impaired cortical connectivity (Sinha et al., 2019). Interestingly, there might be genetic interactions between APOE4 with ABCA7 that impact memory (Chang et al., 2019). ABCA7 expression in the brain is highest in neurons (Zhang et al., 2014).

## BIN1

Bridging integrator-1 gene (*BIN1)* encodes for Bin1, a member of the BAR (Bin–Amphiphysin–Rvsp) superfamily (Sakamuro et al., 1996), homologous to the previously reported amphiphysin-1 (Lichte et al., 1992; Leprince et al., 1997). Bin1, with highest expression in brain and skeletal muscle (Sakamuro et al., 1996; Butler et al., 1997), undergoes alternative splicing (Tsutsui et al., 1997), originating at least 10 isoforms. The main isoforms are a longer neuronal-specific isoform with an extra clathrin binding domain (CLAP), two ubiquitous, and a muscle isoform (Ramjaun et al., 1997). All contain an N-BAR domain, through which Bin1 confers and senses membrane curvature, and a C-terminal SH3 domain that mediates Bin1 interaction with proteins involved in endocytosis, such as amphiphysin-1, dynamin (Butler et al., 1997; Leprince et al., 1997; Ramjaun et al., 1997; Wigge et al., 1997) and endophilin (Micheva et al., 1997). The brain expresses mainly the Bin1 neuronal-specific isoform and at least one ubiquitous isoform (Prokic et al., 2014). Initially, Bin1 was detected in brain synaptosomes and was shown to localize to axon initial segments and nodes of Ranvier (Butler et al., 1997; Wigge et al., 1997; Wigge and McMahon, 1998).

Bin1 can dimerize, through its BAR domain, with itself or with amphiphysin-1 (Wigge et al., 1997). Overexpressed Bin1 inhibits clathrin-mediated endocytosis (Wigge et al., 1997). In contrast, BIN1 knockdown reduced the recycling but not endocytosis of the transferrin receptor in fibroblasts and HeLa cells (Muller et al., 2003; Pant et al., 2009). In neurons, Bin1 polarizes to axons, where its knockdown reduced BACE1 recycling and degradation but not its endocytosis (Miyagawa et al., 2016; Ubelmann et al., 2017). Mechanistically, Bin1 contributes to the scission of recycling carriers from early endosomes (Ubelmann et al., 2017). Neuronal BIN1 knockdown enlarges early endosomes by cargo accumulation or over-activation of Rab5, a small GTPase and regulator of early endosome formation, by interaction with RIN3, a Rab5 GEF and a putative risk factor for LOAD (Calafate et al., 2016).

The expression of Bin1 in the AD brain remains controversial. In AD human brains, *BIN1* transcription increases (Chapuis et al., 2013). In contrast, BIN1 protein decreases in sporadic AD human brains (Glennon et al., 2013). In eFAD models, there is BIN1 accumulation adjacent to amyloid plaques (De Rossi et al., 2019). Subsequently, a separate analysis of two Bin1 isoforms, neuronal and ubiquitous, indicated reduced neuronal *BIN1* but increased ubiquitous *BIN1* in AD human brains (Holler et al., 2014; De Rossi et al., 2016).

To investigate the impact of Bin1 overexpression, the Lambert lab generated a human BIN1 transgenic mouse (h*Bin1*-Tg; Daudin et al., 2018). The hBin1-Tg mice showed an early phenotype of neurodegeneration starting at 3 months, with structural impairment of fiber pathways and structural and functional changes in entorhinal cortex-dentate gyrus (EC-DG) pathway, the earliest brain region impacted in LOAD (Khan et al., 2014; Small, 2014). BIN1 overexpression seems to affect hippocampal physiology, even in the absence of amyloid plaques (Daudin et al., 2018). These data show that the BIN1 risk factor can be sufficient to generate phenotypic changes before the accumulation of Aβ plaques or tau aggregates.

The di Camilli lab identified a synaptic role for Bin1 (Di Paolo et al., 2002). They found Bin1 depleted in the amphiphysin-1 knockout mice. Amphiphysin-1 knockout and *Bin1* knockdown mice synaptosomes exhibited SV recycling defects, such as slower recycling and a smaller pool of SVs and irreversible seizures (Di Paolo et al., 2002). Unfortunately, BIN1 knockout mice die after birth due to muscle defects (Muller et al., 2003). In neurons, Bin1 enriched in axons (Ubelmann et al., 2017) has a presynaptic localization (Di Paolo et al., 2002) and *in vitro* binds to SV glycoprotein 2A (SV2A; Yao et al., 2010), suggesting a presynaptic role for Bin1.

Additionally, a proteomic analysis of postsynaptic compartments identified Bin1, suggesting a postsynaptic role for Bin1 (Bayés et al., 2011). Subsequently, both an increase and a decrease in Bin1 levels led to a small impact on spine morphology (Schürmann et al., 2019). BIN1 knockdown resulted in a reduction of the mean amplitude of AMPAR currents and reduced surface expression of GluA1 in spines and the dendritic shaft (Schürmann et al., 2019). Given that Bin1 is a regulator of endocytic recycling (Pant et al., 2009; Ubelmann et al., 2017), it could be necessary for GluA1 synaptic insertion, *via* recycling carriers from postsynaptic endosomes (Oku and Huganir, 2013).

## CD2AP

CD2AP, CD2-associated protein, belongs to the CIN85/CD2AP protein family, contains three SH3 domains in the N-terminal, a proline-rich domain, and a coil-coiled domain in the C-terminal. CD2AP enriched in podocytes functions in kidney glomeruli (Shih et al., 1999; Dikic, 2002). In podocytes foot processes, in T-cell contacts, at the lamellipodia of migrating cells, CD2AP is at the interface between endocytic trafficking and actin cytoskeleton (Dustin et al., 1998; Li et al., 2000; Welsch et al., 2005; Zhao et al., 2013).

It is still unknown whether CD2AP has a synaptic function. Interestingly, in the kidney, CD2AP interacts with endophilin and, as CIN85, with dynamin and synaptojanin, proteins that have synaptic functions in neurons (Soda et al., 2012). In neurons, a pool of CD2AP localizes to dendritic but not axonal early endosomes (Ubelmann et al., 2017). Functionally, there is only one recent study of Cindr, CD2AP/CIN85 homolog in *Drosophila melanogaster* (Ojelade et al., 2019). Cindr localizes to presynaptic boutons. Importantly, Cindr depletion leads to the impairment of synaptic maturation and SV recycling and release. Mechanistically, Cindr forms a complex with 14–3–3, a regulator of UPS-mediated degradation, to control the levels of calcium channels and synapsin (Ojelade et al., 2019). This study suggests that CD2AP, like Cindr, could regulate neurotransmission and synaptic plasticity.

In the future, it would be imperative to understand if in AD brain CD2AP expression is altered and if its loss of function has an impact on synapses, whether if it is through presynaptic channels degradation or postsynaptic endosomal missorting.

## PICALM

PICALM encodes for CALM (clathrin assembly lymphoid myeloid leukemia), a ubiquitous expressed endocytic clathrin adaptor, the paralog of the neuronal-specific AP180 (Dreyling et al., 1996; Yao et al., 2005). Both bind to membrane lipids, such as PI(4, 5)P2, through their *N*-terminal domain and interact with clathrin through their C-terminal (Yao et al., 2005). In neurons, AP180 is mostly presynaptic, while CALM is both pre- and postsynaptic (Yao et al., 2005). AP180 and CALM are involved in neuronal growth, but are not functionally redundant; AP180 controls axonal development, while CALM controls dendritic morphology (Bushlin et al., 2008). AP180 participates specifically in clathrin-mediated SV recycling (Vanlandingham et al., 2014).

At the plasma membrane, CALM sorts cargo into nascent clathrin-coated vesicles and recruits R-SNARES (Vamp2, 3, 4, 7, and 8) to endocytic vesicles allowing for fusion into early endosomes (Meyerholz et al., 2005; Sahlender et al., 2013).

Presynaptically, CALM facilitates VAMP2 synaptic endocytosis (Harel et al., 2008; Koo et al., 2011; Gimber et al., 2015). Interestingly, VAMP2 is essential for the fusion of SVs and neurotransmitter release (Fernández-Alfonso and Ryan, 2006). CALM can also recluster associated and integral synaptic proteins, such as synaptophysin and synaptotagmin, after SV exocytosis, for sorting into endocytic vesicles for SV recycling (Gimber et al., 2015; Xu et al., 2015).

Postsynaptically, CALM can modulate the abundance of GluA2 at the postsynaptic membrane by influencing its endocytosis (Harel et al., 2011), indicating a possible role of CALM in AMPA receptor dysfunction in AD.

## EPH1A

Eph receptors are a large family of receptor tyrosine kinases (RTK). Based on their structural similarity and the interaction with their ligands, the Eph family members are divided into two types, Eph type-A (EphA) and Eph type-B receptors (EphB), with nine and five members respectively. Their natural ligands are membrane-anchored molecules bound to the cell surface called ephrins. EphrinAs, with higher affinity to EphA, are ligands bound to the cell surface through a glycosylphosphatidylinositol (GPI)-linked moiety, and ephrinBs preferentially interact with transmembrane EphB (Pasquale, 2004; Héroult et al., 2006).

Ephrins also present reverse signaling properties, and Eph-ephrin binding can function in a bidirectional way between two opposing cells. Therefore, Ephs and ephrins localize both pre- or postsynaptically; their signaling can be initiated at the presynapse and move forward or reverse *via* the postsynaptic Eph receptor, and signaling can be initiated at the postsynapse and translated through a presynaptic Eph receptor (Klein, 2009).

Ephs and ephrins function in the formation and regulation of excitatory synapses is well established (Hruska and Dalva, 2012; Sloniowski and Ethell, 2012). EphA4 regulates dendritic spine morphogenesis in the hippocampus, triggering dendritic spine retraction (Bourgin et al., 2007) in a process involving actin (Zhou et al., 2012), Cdk5 (Fu et al., 2007) and integrins (Bourgin et al., 2007). Also, postsynaptic EphA4, but not axonal, is required for synaptic plasticity (Filosa et al., 2009). EphB is necessary for spine formation *via* N-WASP and Cdc42 (Irie and Yamaguchi, 2002), in an RhoA-GEF-dependent manner (Penzes et al., 2003; Margolis et al., 2010). Also, EphB is necessary for spine morphogenesis and synapse formation in the hippocampus (Henkemeyer et al., 2003), as well as for the recruitment, localization, and function of NMDA receptors (Dalva et al., 2000; Takasu et al., 2002; Nolt et al., 2011). EphBs are also crucial for synaptic plasticity (Grunwald et al., 2004).

In AD-related models, Aβ oligomers can bind to EphB2, depleting them, and consequently impairing synaptic plasticity and cognitive functions (Cissé et al., 2011).

Although the EPHA1 gene has been known as a putative risk factor since 2011 (Hollingworth et al., 2011; Naj et al., 2011), it is still unclear how EPHA1 could contribute to LOAD synapse dysfunction.

## SORL1

SORL1 encodes for the sortilin-related receptor with A-type repeats (Sorla), also known as LR11, a member of the vacuolar protein sorting ten domain (VPS10) receptor family and LDLR family (Jacobsen et al., 1996; Yamazaki et al., 1996). SORL1 variants were first associated with LOAD in 2007 (Rogaeva et al., 2007), but a decrease in Sorla expression in the brain of AD patients had been already observed in 2004 by the Lah lab (Scherzer et al., 2004) and in 2005 by the Willnow lab (Andersen et al., 2005). Even in mild cognitive impairment, there is less Sorla expression (Sager et al., 2007). Sorla expression is widespread in the brain (Motoi et al., 1999) and localizes to early endosomes and Golgi compartments (Andersen et al., 2005; Offe et al., 2006) as well as pre- and postsynaptic compartments (Rohe et al., 2013; Hartl et al., 2016).

SORL1’s role in synaptic function is unclear. In neurons, Sorla may function as a neuroprotective factor supported by findings that Sorla is a sorting factor for the tropomyosin-related kinase receptor B (TrkB) to facilitate trafficking between synaptic plasma membrane, postsynaptic densities, and cell soma (Rohe et al., 2013). TrkB is a receptor of brain-derived neurotrophic factor (BDNF), a growth factor involved in neuronal survival (Chao, 2003).

Presynaptically, Sorla can interact with phosphorylated synapsin, *via* 14–3–3 adaptor proteins to sort it for degradation by the protease calpain (Hartl et al., 2016). Also, supporting a role for Sorla in regulating synapsin turnover, a proteomic study of cortices and hippocampus of SORL1-deficient mice revealed an increase in synapsin 1 and 2 (Hartl et al., 2016).

Postsynaptically, Sorla interacts with the synaptic EphA4 receptor tyrosine kinase attenuating its activation by ephrinA1 (Huang et al., 2017). Interestingly, EPhA4 activation by ephrinA1 induces spine retraction (Sloniowski and Ethell, 2012). Sorla overexpression *in vivo* decreases EPhA4 activation and reduces the deficits caused by Aβ in LTP and memory (Fu et al., 2014; Huang et al., 2017). This study indicates that in LOAD, decreased Sorla may account for increased activation of spine retraction, maybe even in the absence of Aβ.

**Figure 3:**
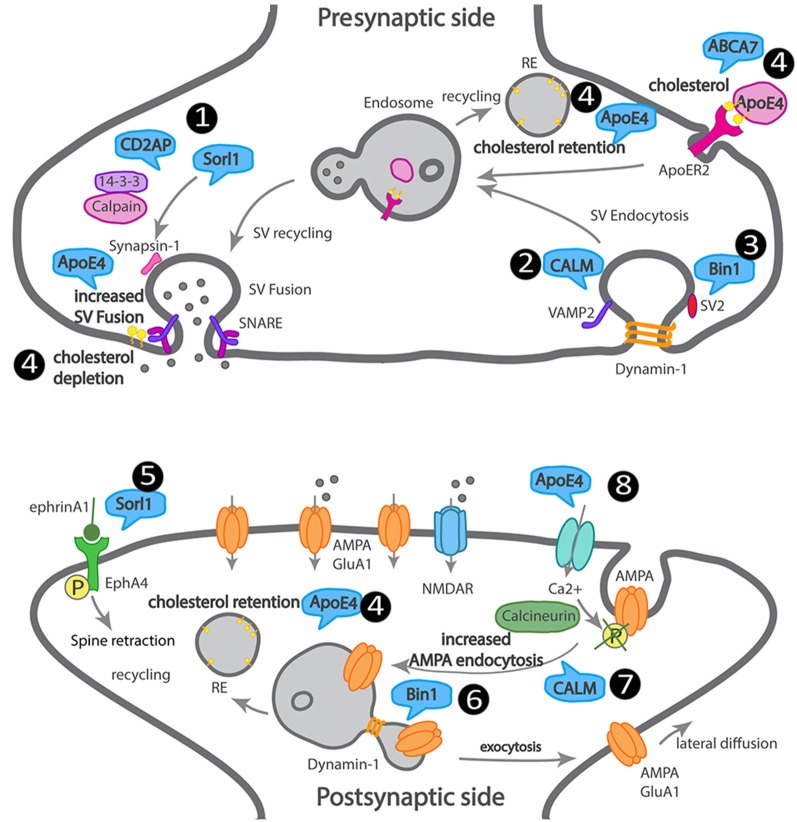
Scheme of a dysfunctional LOAD synapse illustrating how LOAD trafficking genes impact synaptic trafficking. At the presynaptic terminal, 1. Sorl1 and Cindr (CD2AP homolog) interact with synapsin, *via* 14–3–3, and their loss of function leads to aberrant synapsin accumulation. 2. CALM mediates VAMP2 endocytosis and SNARE complex recycling. 3. Bin1 interacts with dynamin and SV2 and could play a role in SV endocytosis. 4. ApoE4 and ABCA7 alter the transport of cholesterol and other lipids into neurons, leading to cholesterol retention in recycling endosomes and depletion from the synaptic membranes. At the postsynaptic terminal, 5. Sorl1 attenuates EphA4 activation by ephrinA1, causing spine retraction. 6. Bin1 regulates GluA1 availability at synapses *via* recycling. 7. CALM regulates GluA2 availability at synapses *via* endocytosis. 8. ApoE4 regulates AMPA receptor availability at synapses *via* calcium influx, calcineurin activation, that dephosphorylates AMPA receptors triggering their endocytosis.

We attempted to summarize how LOAD trafficking genes may affect synapses, although direct supporting data is missing in most cases (Figure 3).

## Conclusion

For many years, LOAD etiology has been a debatable topic among researchers. The amyloid hypothesis has dominated the field as the leading cause of AD. Experimental models of eFAD support this hypothesis, as well as the delay of disease onset in carriers of the AD protective mutation in APP that reduces Aβ production (Jonsson et al., 2012). It remains unclear if Aβ accumulation causes the earliest synaptic dysfunction in LOAD.

Moreover, most clinical trials targeting Aβ aggregation cleared amyloid plaques, but the cognitive improvement was limited at best. Today, clinical trials include patients early in the disease, but it is still unclear if once disease manifests, it will be possible to revert synapse damage. In the future, based on the genetic risk, it will be possible to predict the age of onset, and thus preventive therapeutic strategies targeted at the still reversible synapse dysfunction may be the solution. Now, we need to determine the targets for future preventive therapies based on the mechanisms that drive the initial synaptic dysfunction in AD.

Here we reviewed the Aβ-dependent mechanisms of synapse function and dysfunction as well as potentially Aβ-independent mechanisms. We found evidence in the literature supporting that Aβ, at the physiological concentration (picomolar), facilitates synaptic transmission and plasticity while reducing it when at pathological levels (nanomolar). Pathological Aβ has multiple synaptic targets indicating a broad impact on synapse or a lack of specificity. Alternatively, the multiplication of Aβ targets reflects the need for an effort from the research community to improve cellular and *in vivo* models, to uniformize the experimental conditions, and to integrate individual findings. Overall, we find that presynaptically, Aβ targets the synaptic vesicle cycle and neurotransmitter release. However, the mechanism thus far identified depends on the disruption of the endosomal membrane by Aβ. Once at the cytosol, at least *in vitro*, Aβ can bind to the SV release machinery and disrupt SV release. Postsynaptically, Aβ targets spine loss and glutamate receptors endocytosis, depressing synaptic transmission; the mechanism involved seems to depend on extracellular Aβ-driven calcium influx and endosomal Aβ. However, the mechanisms by which endosomal Aβ interferes with synapses remain mostly unknown.

Moreover, the generalized use of exogenous Aβ treatments or transgenic models overexpressing APP or presenilin with eFAD mutations may disguise relevant Aβ-dependent synaptic dysfunction mechanisms, such as the ones mediated by aging or by LOAD risk factors. So, there is an increasing need for better models to understand the physiological targets of Aβ in AD.

Regarding Aβ-independent mechanisms of synapse dysfunction, we focused on LOAD genetic risk factors linked to endocytic trafficking, given its importance for synapses. There are few studies on the impact of these genes’ loss of function and even less on the effects of patient’s variants. Nevertheless, it is interesting to find that most of the LOAD genes, similarly to Aβ, control the synaptic vesicle cycle and the trafficking of glutamate receptors.

Knowing the synaptic dysfunction mechanisms used by each LOAD gene will likely reveal novel targets that may be independent of Aβ. It would be essential to correlate the mechanism of each LOAD gene with the cognitive decline and the carrier genotype.

Therefore, new preclinical animal and cellular models bearing LOAD genetic risk are being established and will hopefully soon contribute to identifying early LOAD therapeutic targets and potential biomarkers. Thus, with these new medical strategies, we might approach LOAD during its prodromal phase, and we might minimize the disease burden, expanding life span with a better quality of life.

## Author Contributions

CP: review of presynaptic mechanisms and LOAD genetic risk factors presynaptic (dys)functions. MB: Go analysis of LOAD genes and preparation of Figure 1. MB and JC: introduction. MB, JC, and MA: review of Abeta synaptotoxicity. MA: review of postsynaptic mechanisms and LOAD genetic risk factors postsynaptic (dys)functions. FM: illustration of Figures 2, 3. CP and FM: writing modifications and feedback. CG: supervision, writing, interpretation, and editing of the manuscript.

## Conflict of Interest

The authors declare that the research was conducted in the absence of any commercial or financial relationships that could be construed as a potential conflict of interest.

## Footnotes

^1^https://www.alzforum.org/alzgene
